# Relationship between Nest and Body Temperature and Microclimate in the Paper Wasp *Polistes dominula*

**DOI:** 10.3390/insects14110886

**Published:** 2023-11-16

**Authors:** Helmut Kovac, Julia Magdalena Nagy, Helmut Käfer, Anton Stabentheiner

**Affiliations:** Institute of Biology, University of Graz, 8010 Graz, Austria

**Keywords:** paper wasp, *Polistes dominula*, nest temperature, body temperature, thermoregulation, climate, microclimate

## Abstract

**Simple Summary:**

The heat-loving paper wasp *Polistes dominula* builds small nests in sheltered places in quite differing habitats, providing a favorable microclimate for brood development. Their careful choice of the nesting site enables them to keep the nest and brood temperature above the ambient air temperature. In temperate Austrian (Central European) climates, the cooling efforts of the adult wasps keep the mean comb temperature from exceeding approximately 39 °C, which seems to be a threshold for optimal development. Measurement of the nest and the body temperature of the wasps at typical nesting sites revealed a close relationship with the air temperature at the nests but a suboptimal correlation with climate data from a local standard weather station or model-generated macroclimate data. The comparison emphasizes the importance of microclimate measurements if it is to judge the vulnerability of insects to climate change.

**Abstract:**

The paper wasp *Polistes dominula* is a thermophilic species originating from the Mediterranean climate, but is now widely spread in Europe. They live in quite differing habitats; and as synanthropic species, they have been established in human settlement areas. They build a single small comb at protected places with a favorable microclimate. We measured the temperature of the wasps, the nests and their environment at typical nesting sides in Austria (Europe) in the temperate climate, in order to reveal relationships between nest and body temperature and the habitats’ microclimate. The temperatures of the comb and of the wasps’ body were in a wide range (~20–37 °C) above the ambient air temperature at the nest. This is an advantage as higher temperatures accelerate the development speed of the brood. However, the mean comb temperature did not exceed approximately 38.6 °C. This was managed by cooling efforts of the adult wasps. The ambient air temperature near the nest (~1–2 cm) was always clearly elevated above the ambient air temperature at a local standard weather station in the habitat. A comparison with climate-model-generated macroclimate data revealed the necessity of measuring microclimate data for a reliable description of the insects’ thermal environment.

## 1. Introduction

The paper wasp *P. dominula* is a thermophilic species originating from the Mediterranean climate region in Europe, but is now widely spread in Europe and—as an invasive species—also on other continents. *P. dominula* has expanded its distribution range immensely and has settled in regions with harsher climatic conditions and even survives in alpine regions at medium altitudes in Central Europe at sheltered places provided by human settlements. Nowadays, it is an abundant *Polistes* species in Europe [1]. Its distribution covers southern and central Europe; and possibly due to climate change, it is still expanding its range to the north and has reached Northern Germany and Denmark [2,3,4,5]. They live in quite differing habitats and have established themselves as a synanthropic species, preferably in human settlement areas.

In spring, a foundress queen builds a single small comb at protected places with a favorable microclimate. The building material is chewed wood fibers mixed with saliva from the wasp, a material which is similar to paper. The comb affords some protection for the brood, but it has to be sheltered from rain.

In many insects, the development of the brood depends on ambient temperature and extreme variations in temperature could be detrimental (see also [6]). Many social insect species are able to control and regulate the temperature within their nests with active and passive mechanisms of thermoregulation. In honeybees, a species with distinct endothermic abilities, heat production and insulation of the breeding cavity enable the bees to regulate the brood nest temperature within the narrow range of 32–36 °C (e.g., [7,8,9]). Some vespine wasps exhibit endothermic behavior during foraging and other activities (e.g., [10,11,12]), and they are able to control and regulate the temperature within their nests, but not with an accuracy like honeybees [13,14,15]. Polistine wasps, by contrast, exhibit only a weak endothermic performance. Endothermic heat production was observed in preparation for flight, during foraging and nest defense [12,16,17,18,19]. Active mechanisms for nest thermoregulation like clustering and generating metabolic heat or active heating of single individuals, i.e., the direct incubation of a brood cell, has not been observed. Heat production on the nest would not be very effective, as the nests consists of just a single comb without any protecting shell. The produced heat would be lost to the environment immediately. However, responses to high temperatures like wing fanning, or evaporative cooling by water droplets to prevent overheating of the brood are very well known (e.g., [20,21,22,23]). Due to this fact, the thermal conditions at the nests of temperate-zone polistine wasps depends predominantly on the environmental conditions at the nesting sites. Passive mechanisms of nest thermoregulation, including all mechanisms which help to optimize the nest temperatures, like site selection or special structures of nests, are of significant importance. In comparison to open-nesting species like *P. biglumis* and *P. gallicus* (e.g., [17,22,23,24,25])), the nest site choice at warm sheltered nesting sites of *P. dominula* enables them to achieve a nest microclimate in the temperate climate of Central Europe not much different from that of *P. gallicus* nesting in the open in the warmer Mediterranean climate [18]. Therefore, we compare our data with similar measurements on the related *P. gallicus* [23], where the distribution range is mainly limited to its original Mediterranean climate region.

The distribution range of *P. dominula* covers southern and central Europe, and part of Northern Europe [2,3,4,5] with considerably differing habitats. Such species with large distribution ranges provide powerful study systems for understanding adaptation to different environmental conditions, revealing knowledge about adaptations on highly variable thermal conditions. Their success across variable environments makes them suitable models for exploring which traits will be important for resilience to climate change, because temperature is a crucial factor in this context. In an attempt to broaden the present knowledge [17] on the ability of this species to cope with microclimatic variation, we focused on the thermal conditions at typical nesting sites in the temperate climate of Central Europe. We measured the temperature of the wasps and the nests, and of their microclimatic environment in a typical habitat in order to generate mathematical descriptions (functions) of the relationships between these parameters. These functions aim at future generation of energy consumption models of the wasps’ brood during the breeding season (compare [26]). A comparison with climate-model-generated macroclimate data (INCA_L, GeoSphere Austria [27]) should show how well such large-scale climate data are suited to estimate the body temperature of these wasps and their brood (on this topic, see also [28]). The comparison of inhabited and abandoned nests should show differences in comb temperatures and reveal the wasps successful thermoregulatory activity. Our data provide the basics for model calculations of the environmental conditions in the wasps’ habitat under future climate conditions. Such data, considering the actual microclimate, are indispensable to predict chances of survival and further distribution of this species in an ongoing changing world.

## 2. Materials and Methods

### 2.1. Research Location and Wasps

**Figure 1:**
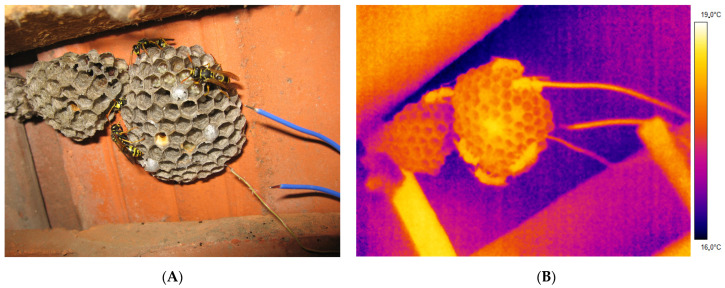
Inhabited (**right**) and abandoned nest (**left**) of *Polistes dominula* attached to a roofing tile. (**A**) photograph; (**B**) infrared thermogram.

The research location was in a habitat typical for the paper wasp *P. dominula*, in a rural area in Gschwendt (Styria, Austria) in the temperate climate region of Central Europe, where a stable population of the paper wasps has been observed continuously for decades (Figure S1). The nests were located at very typical nesting sites in the loft of a farmhouse, and in a rather untypical location, in an old car tire. Measurements were conducted on 10 inhabited and 7 abandoned nests. The abandoned nests (old nests from the last year) were always close to the inhabited nests and therefore could be measured simultaneously with the inhabited nests (Figure 1A). Measurements were conducted in summer 2017 (June–August) at six nests, and in spring 2018 (May–June) at four nests (Table S1). In spring, only foundress queens were on the nests. In summer, workers were also present at the nests.

### 2.2. Measurement of Body, Nest and Air Temperature

The surface temperature of the comb, wasps (head, thorax, abdomen) and substrate (to which the nest was attached to) was measured by infrared thermography, without touching and impairing the wasps or the nests, with a FLIR T650sc (FLIR Systems Inc., Wilsonville, OR, USA) infrared camera (resolution 640 × 480 pixels, sensitivity < 20 mK). The measurement accuracy was approximately 0.7 °C, assuming a wasp cuticle infrared emissivity of 0.97 [23], a nest material emissivity of 0.94, and a substrate (stone or concrete) emissivity of 0.93 for the nest background. Infrared data were stored digitally on internal memory cards and evaluated later in the laboratory. Measurements were conducted continuously at least for 12 h, but at several times for 24 h. The infrared thermograms (Figure 1B) were stored at a rate of 1/min, but evaluation of the thermograms was performed in 10 min intervals. Evaluation of the surface temperatures of the wasps and the combs was performed with FLIR ThermaCam Researcher Pro 2.10 (FLIR Systems Inc., Wilsonville, OR, USA), controlled by a custom programmed Excel (Microsoft Corporation, Redmond, WA, USA) VBA macro which also extracted the microclimatic data from the logger files at the exact time of thermographic measurement. For the measurement of the mean nest temperature, an outline of the comb was drawn with tools of the infrared software, and the average of the enclosed pixels calculated. The temperature of the body parts was measured with square tools, which covered a large portion of the body part. For further details of temperature evaluation, see also [23]. We analyzed the comb and body temperature in relation to the ambient air temperature, temperature of a standard weather station and model-generated macroclimate data.

### 2.3. Measurement of Micro- and Macroclimate

The microclimate at the nests and the macroclimate (local climate) in the habitat were measured continuously during the entire investigation period. The air temperature at the nests (T_a_nest) was measured with NiCr/Ni thermocouples (OMEGA Engineering, Stamford, USA), close to the combs (1–2 cm, Figure 1A) and stored with data-loggers (ALMEMO 2690, Ahlborn GmbH, Holzkirchen, Germany) at 1/s intervals. A standard meteorological weather station (T_standard_) in the habitat (approximately 20 m from the nests) recorded meteorological data (temperature, humidity, radiation, wind speed) continuously at 10 min intervals during the two years of investigations. For comparison, large-scale macroclimate data sets were obtained by ZAMG’s homogenized high resolution network of weather stations in Austria (INCA: 1 km × 1 km grid, hourly resolution, ensemble data) via the ZAMG data hub [27].

### 2.4. Data Analysis and Statistics

All data evaluations and calculations were performed with MS Excel (Microsoft Corporation, Redmond, WA, USA). Curve plots were performed with Origin 2017 software (version 94G, b 9.4.0.220OriginLab Corporation, Northampton, MA, USA), and the accompanying statistics was generated with Statgraphics software (Statgraphics Centurion XVI, StatPoint Technology Inc., The Plains, VA, USA). Multiple regression statistics (relationship of nest and wasp body temperatures) and multifactor ANOVA (to test for differences of nest and body temperature control between species after compensation of environmental variables) were performed with Statgraphics software. We present the comb and body temperature in relation to the air temperature at the nest (T_a_nest) or the local habitat’s air temperature (T_a_standard). We created data for a “simple temperature relationship”, where the investigated parameter is assumed to depend linearly on ambient air temperature (isothermal line with a slope of 1; T_comb_ = T_a_nest, T_thorax_ = T_a_nest), and conducted an ANOVA to compare the deviation of the observed from the calculated data. Analysis of comb and thorax temperatures showed that they could be best described by polynomial functions. We determined the points of intersection of comb and thorax temperature with the isothermal line. The intersection of the two lines is the point where regulatory behavior had an effect strong enough to cool the comb or body. Statistical details are provided in the Supplementary Information (Tables S2–S10).

## 3. Results

**Figure 2:**
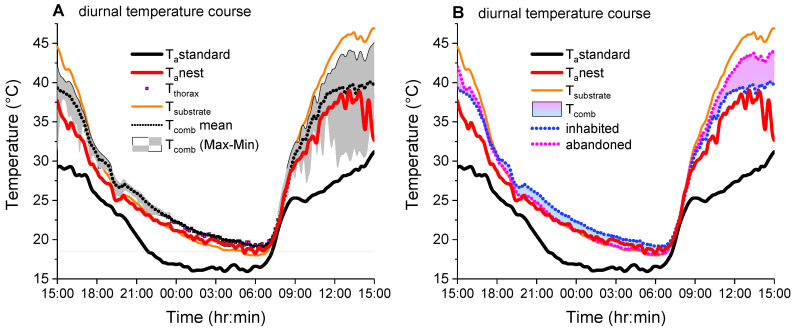
Diurnal temperature variation at a nest of *Polistes dominula* in Gschwendt (Austria). (**A**) Inhabited nest; (**B**) abandoned nest. T_thorax_ = the mean thorax surface temperature of a wasp; T_comb_ = the mean surface temperature of the comb, gray ribbon: total range of comb temperature (T_max_:T_min_); T_a_nest = ambient air temperature nearby the nest (1–2 cm); T_a_standard = the ambient air temperature of a standard meteorological weather station (approximately 20 m away from the nest). T_substrate_ = roofing tile temperature beside the nest.

Measurement of the comb, body and air temperature showed considerable diurnal temperature variation in these parameters (Figure 2). The temperatures increased by day and decreased at night. The comb and thorax temperature curves followed the ambient air temperature at the nest (T_a_nest) much closer than the ambient air temperature of the local weather station (T_a_standard). While at night temperatures differed little between different parts of the nest, temperatures varied considerably during daytime (gray Max–Min band in Figure 2A). The temperature of the substrate where the nests were fixed to was mostly higher than the nest temperatures during daytime, especially on sunny days, but lower at night.

### 3.1. Comb Temperature

**Figure 3:**
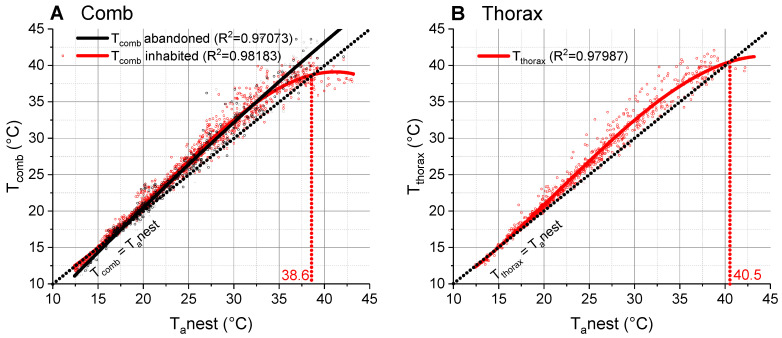
Comb and thorax temperature in relation to ambient nest temperature (T_a_nest): (**A**) Comb temperature (T_comb_) of inhabited and abandoned nests. (**B**) Thorax temperature (T_thorax_) of wasps. from inhabited nests. Dotted isothermal lines indicate a slope of 1 (where T_comb_ or T_thorax_ is equal to T_a_nest). The points of intersection with isothermal lines (T_comb_ = T_a_nest; T_thorax_ = T_a_nest) are indicated by vertical dotted lines.

At lower temperatures (<20 °C), the surface temperature of the abandoned and inhabited combs was quite similar to the ambient air temperature at the nest. At higher temperatures, some deviation above the ambient air was observed, with a maximum mean elevation of approximately 2.5 °C (Figure 3A). The temperature of the abandoned nests increased linearly with the ambient air even at the highest T_a_nest. By way of contrast, the temperature of the inhabited nests followed a polynomial curve and crossed the isothermal line at 38.7 °C. Above this threshold, the comb temperature remained below the ambient air temperature. Both the temperature of the inhabited and the abandoned combs differed significantly from the isothermal line (*p* < 0.0001, ANOVA) and they differed also significantly from each other (*p* < 0.0001, ANOVA). Fit functions and parameters are given in Table S2.

**Figure 4:**
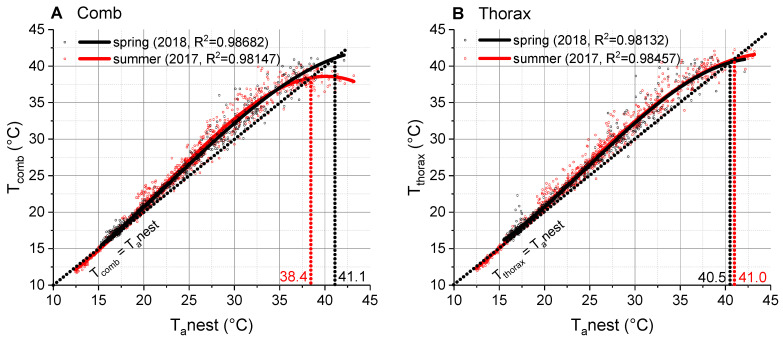
Comb and thorax temperature in relation to ambient nest temperature (T_a_nest): (**A**) comb temperature (T_comb_) in spring (2018) and summer (2017); (**B**) thorax temperature (T_thorax_) of wasps in spring (2018) and summer (2017). Dotted isothermal lines indicate a slope of 1 (where T_comb_ or T_thorax_ is equal to T_a_nest). The points of intersection with isothermal lines (T_comb_ = T_a_nest; T_thorax_ = T_a_nest) are indicated by vertical dotted lines.

**Figure 5:**
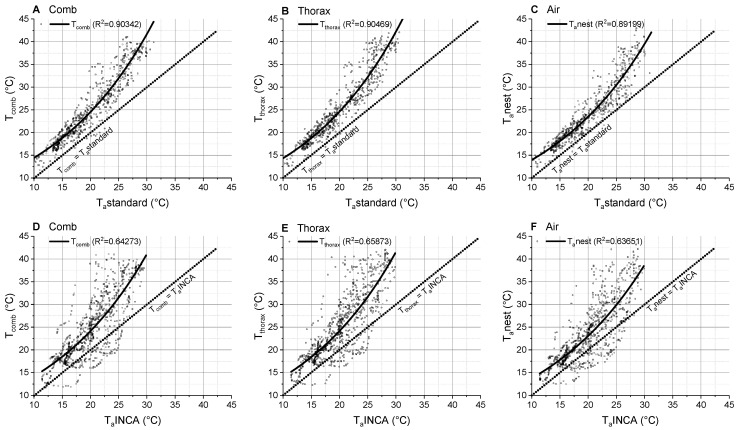
Comb temperature (T_comb_), thorax temperature (T_thorax_) and ambient nest temperature (T_a_nest, 1–2 cm beside the nests), in relation to (**A**–**C**) the standard ambient temperature of a local weather station (T_a_standard, approximately 20 m away from the nest), and (**D**–**F**) in relation to INCA weather data of GeoSphere Austria (T_a_INCA). Dotted isothermal lines indicate a slope of 1 (where T_comb_, T_thorax_ or T_a_nest is equal to T_a_standard or T_a_INCA).

**Figure 6:**
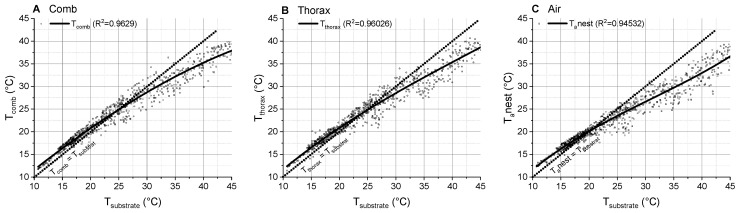
(**A**) Comb temperature (T_comb_), (**B**) thorax surface temperature (T_thorax_), and (**C**) ambient nest temperature (T_a_nest, 1–2 cm beside the nest), in relation to substrate temperature where the nest was attached to (T_substrate_). Dotted isothermal line indicates a slope of 1 (where T_a_nest, T_comb_ or T_thorax_ is equal to T_substrate_).

Comparison of the seasonal temperatures of the inhabited combs in spring and summer showed that they were quite similar but differed in the points of intersection with the isothermal line (spring: 41.1 °C, summer: 38.4 °C; Figure 4A). Temperature data of edge cells (empty) and center cells (with brood) were also quite similar but had also different points of intersection with the isothermal line (edge: 39.0 °C, center: 38.3 °C; Figure S3). A comparison of the ambient air temperature at the nest with the air temperature measured at the local standard meteorological weather station revealed a non-linear relationship. The T_a_nest was clearly higher than the T_a_standard and increased in a simple exponential course (Figure 5C), similar to the comb temperature (Figure 5A). Both the comb temperature and T_a_nest correlated closely with T_substrate_ but remained below it at higher substrate temperatures (Figure 6). Fit functions and parameters are given in Table S2.

### 3.2. Body Temperature

As the wasps were mainly ectothermic, i.e., the temperature of the three body parts was very similar, we focus on the temperature of the thorax (Figure 3B; compare Figure S4). Up to 20 °C, the surface temperature of the thorax resembled the ambient air temperature at the nest (T_a_nest) and increased with rising temperature in a polynomial course (Figure 3B). At higher temperatures, it was higher than T_a_nest and showed a maximal mean deviation of approximately 2.5 °C. The point of intersection with the isothermal line was at 40.5 °C. Above this threshold, the thorax temperature remained below the ambient air temperature. The thorax temperature differed significantly from the isothermal line (*p* < 0.0001, ANOVA). The wasps had a very similar thoracic temperature in spring to that of wasps in summer (Figure 4B), just the point of intersection with the isothermal line differed by 0.5 °C (spring: 40.5 °C, summer: 41.0 °C). Fit functions and parameters are given in Table S2.

### 3.3. P. dominula vs. P. gallicus

**Figure 7:**
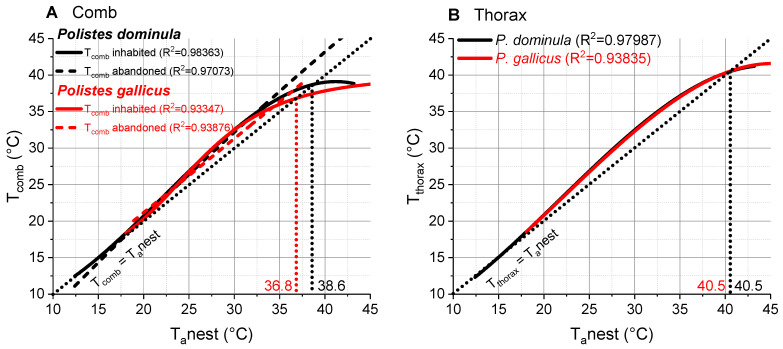
Comb and thorax temperature in relation to ambient nest temperature (T_a_nest): (**A**) comb temperature (T_comb_) of inhabited and abandoned nests of *P. dominula* and *P. gallicus* (modified data from [23]); (**B**) thorax temperature (T_thorax_) of *P. dominula* and *P. gallicus* (modified data from [23]) from inhabited nests. Dotted isothermal lines indicate a slope of 1 (where T_comb_ or T_thorax_ is equal to T_a_nest). Points of intersection with isothermal lines (T_comb_ = T_a_nest and T_thorax_ = T_a_nest) are indicated by vertical dotted lines.

The comparison of the comb temperatures revealed differences between the two species (Figure 7A). In the inhabited combs, the points of intersection with the isothermal line were 38.7 °C in *P. dominula* (this paper) and 36.8 °C in *P. gallicus* (data from [23]). The fit curves of the two species’ combs differed significantly in the slopes (*p* < 0.0001, ANOVA), but not in the intercepts (*p* > 0.05, ANOVA). The abandoned combs differed significantly in both (intercept: *p* < 0.01, slope: *p* < 0.0001, ANOVA). The thorax temperatures of the two species were very similar. The points of intersection with the isothermal line were determined at 40.5 °C in *P. dominula* as well as in *P. gallicus* (Figure 7B). Fit functions and parameters are given in Table S2.

## 4. Discussion

We measured the comb and body temperature and the microclimate at the nests of *P. dominula* in a typical habitat of the temperate Central European climate, in order to explore the relationship between these parameters. At lower temperatures (<20 °C), we observed comb temperatures very similar to the ambient air temperature near the nest (T_a_nest) (Figure 2 and Figure 3A). This is not surprising as polistine paper wasps exhibit no active endothermic heat production for nest thermoregulation (our own unpublished observation, and [21]). At higher environmental temperatures, temperatures of both inhabited and abandoned nests were significantly higher than T_a_nest (compare with isothermal line in Figure 3A). In the abandoned nests, the comb temperature increased linearly with T_a_nest. This finding indicates that the combs got some heat from the environment. The measurement of the substrate (e.g., roofing tiles), where the nests were attached to, revealed that this substrate was much warmer (especially on warm days with sunshine) than the air near the nests and the nests themselves (Figure 2). The complex relationship between temperatures of substrate, air and comb is displayed in Figure 6. The combs gained heat of the substrate and with this heat their temperature was increased above T_a_nest. However, the combs did not reach the temperature of the substrate. This was accomplished by the special linker device between substrate and comb, the pedicel. Höcherl et al. [21] suggested that the pedicel has an important influence on the nest temperature. During hot days, the gap between the comb and the substrate provides a physical insulation of the nest, so that the comb does not reach as high temperatures as the substrate.

In the inhabited nests, the conditions were similar like in the abandoned nests. In both cases, the heat gain from the warm substrate increased the comb temperature above T_a_nest up to a maximum of 2.5 °C. The additional metabolic heat production of larvae and pupae seems to have only a small effect (Figure 3A; compare Figure 2). The lower comb temperatures of the inhabited nests at T_a_nest > ~33 °C, crossing the isothermal line at 38.7 °C (Figure 3A), is accomplished by active thermoregulatory behavior of the adult wasps [21]. For this purpose, the wasps collect water and spread it on the combs. With wing fanning and additional evaporative cooling by the water droplets they cool the comb surface and avoid overheating of the brood. *P. dominula* flexibly uses the same basic behavioral repertoire for nest temperature control than the Alpine *P. biglumis* and the Mediterranean *P. gallicus* [23]. The behavioral and physiological flexibility of *P. dominula* is pointed out by the finding that they are even able to nest in Alpine climate by using human-made sheltered places (Figure S2).

As thermoregulation of the comb is achieved by cooling efforts of the wasps, we were interested whether there are differences between spring and summer. In spring, ambient temperatures are generally lower and there was only the foundress queen at the nest (one additional queen in one case). On sunny days, however, the temperature may nevertheless become so high that thermoregulatory measures are necessary. In addition to fanning, evaporative cooling with water droplets becomes important [18,21,23]. To collect water, the queen has to leave the nest. We suggest that the much higher point of intersection with the isothermal line in spring (41.1 °C) than in summer (38.4 °C) (Figure 4A) reflects the single wasps’ difficulty of proper thermoregulation on hot days in spring. Hot times in spring may therefore limit breeding success in these wasps in such habitats, especially at increasing temperatures due to climate change.

In *P. gallicus*, a closely related species in the Mediterranean climate (Figure 7A, modified data from [23]), up to approximately 32 °C the temperature course of the comb was very similar to that in *P. dominula* from the temperate climate (present study). At higher ambient temperatures, the comb temperature of *P. gallicus* was even lower than in *P. dominula*. The point of intersection with the isothermal line was 38.7 °C in *P. dominula* and 36.8 °C in *P. gallicus*. This is somewhat surprising as one would expect higher (nest) temperatures in the Mediterranean climate. It has to be considered that the Mediterranean species has to deal with higher (absolute) ambient temperatures and therefore they probably need a lower safety margin to cope with detrimental high temperatures. Therefore, we presume that they start with thermoregulatory measures at lower temperatures. In the temperate climate, the foundresses of *P. dominula* build the combs at sheltered places with a favorable microclimate, which can significantly deviate from the temperature in the habitat (Figure 2 and Figure 5). This selection of the optimal nesting site is reflected in a microclimate comparable with that of the Mediterranean species *P. gallicus* nesting in the open in their habitat [18]. Finding suitable nesting sites is very important, especially for species that have only a limited capacity for regulation of the nest temperature. The selected nesting site and thus thermal environment is of benefit for the development of the brood. In the harsher Alpine climate, the alpine species *P. biglumis* builds its nests preferably on rocks, oriented toward east-south-east to gain solar heat of the morning sun. This heat gain increases the brood temperature considerably above the ambient air on sunny days, which speeds up brood development [23].

In open-nesting *Polistes* species, it was found that they built short empty cells at the periphery and longer cells in the center of the nest (*P. chinensis*: [29]; *P. biglumis*: [24]). Yamane et al. [30] interpreted that to be an adjustment to a colder climate. They suggested that this structure may serve as an air chamber similar to the envelope of nests of Vespine wasps. However, at low and moderate T_a_nest, we measured quite similar temperatures in the cells at the edge (without brood) and the center of the combs, where brood (larvae or pupae) mainly was present (Figure S3). From a thermodynamic point of view, it seems unplausible that some empty peripheral cells can have much effect on nest temperature if both the large front and the rear of the nest are exposed to the environment. The slightly lower edge temperatures at high T_a_nest (Figure S3) indicate that the wasps take special care to prevent overheating of the brood. This is similar to *P. gallicus,* where the adults avoid the temperature of the brood to exceed approximately 39 °C (on average) even at the highest environmental temperatures [23].

The thorax temperature increased with ambient temperature in a similar way as the comb temperature, being significantly elevated above the air temperature above 15 °C (Figure 3B). We suggest this to be not only a result of the heat gain from the warm substrate and the nest but also of the metabolic heat production of the wasps themselves. The exponential increase in their resting respiratory metabolism with ambient temperature [18,26,31] will contribute disproportionately to their body temperature elevation at high air temperatures.

The thorax temperature course of *P. dominula* in spring was very similar to that in summer (Figure 4B). Surprisingly, thorax temperature data of the Mediterranean species *P. gallicus* (modified data from [23]) were very similar to our data. The point of intersection with the isothermal line of 40.5 °C in *P. dominula* was identical with that of *P. gallicus* from the warmer climate (Figure 7B). Although the critical thermal maxima (CT_max_) are much higher in both species (*P. dominula*: 47.4 °C, *P. gallicus*: 47.7 °C; [18]), the wasps obviously try to avoid body temperatures higher than approximately 42.5 °C in both climates. Our measurements show that they begin with thermoregulatory measures at temperatures considerably below this critical temperature threshold. The intersection of the isothermal line and the body temperature course is the point where regulatory behavior had an effect strong enough to cool the body to ambient temperature. This regulatory behavior, however, starts already at considerably lower temperatures (~35–37 °C in Figure 4B).

Our investigation revealed the great importance of microclimate measurements to estimate the insects’ body temperature and energy turnover from environmental data and predict changes in future climate scenarios. The temperature of the comb and of the wasps’ thorax depended mainly on the air temperature measured in close vicinity to the nest (T_a_nest) (Figure 3, Figure 4 and Figure 5, Tables S3 and S4). Due to the nest choice of the foundress queen at favorable warm locations, the comb temperature and adult wasps’ body temperatures as well as the ambient temperature at the nest deviated considerably from standard meteorological measurements even of a local weather station (Figure 5). The much weaker relationship of comb, thorax and air temperature with model-generated macroclimatic data of the habitat (T_a_INCA_L) (Figure 5D–F) revealed the inaccuracy of such data (indicated by a lower F-ratio, which represents the accuracy of the fit function, in Tables S3 and S4). This finding once more points out the necessity of measuring microclimatic data for reliable results (see also [32,33,34,35,36,37,38,39,40,41]. With our data, we strongly confirm the statement of Pincebourde and others [42,43], who emphasized the importance of obtaining fine-scale temperature records near any surface of relevance for the investigated animal to explore their habitat’s true environmental conditions. They point out that the major pitfall in distribution modelling is that the actual climatic conditions experienced by organisms in their microhabitat and across their home range are largely ignored [44]. The concept of “microclimate recordings” has to be applied to natural systems to detail the abiotic conditions experienced by organisms in their microhabitat [45,46,47]. Such investigations are particularly important in ectothermic insects, since their body temperature mostly corresponds to the ambient temperature. In Polistine wasps, this is especially important in those species nesting in sheltered places like *P. dominula* and (occasionally) *P. nimpha* (our own observation).

## 5. Conclusions

We found a direct relationship between the comb (brood) and body temperature of the adults with the microclimate at the nesting sites of the paper wasp *P. dominula* in the temperate climate of Central Europe. The temperature of both may strongly deviate from standard meteorological measurements and model-generated macroclimatic data. In the favorable, warm locations chosen for nesting, the thermoregulatory behavior of the wasps prevents overheating to detrimental high temperatures. Our data provide the basic information for model calculations of the nest’s microclimate under current and future climate conditions. These data are indispensable to predict the survival chances in the current habitats and the distribution of the species in a changing environment.

## Data Availability

All data are available in the manuscript or in the Supplementary Materials.

## Supplementary Materials

The following supporting information can be downloaded at: https://www.mdpi.com/article/10.3390/insects14110886/s1, Table S1: Nest statistics for temperature measurements; Table S2: Statistics, fit functions and parameters for temperature measurements; Table S3: Multiple linear regression model and ANOVA of comb temperature; Table S4: Multiple linear regression model and ANOVA of thorax temperature; Table S5: Comparison of regression lines and ANOVA of comb temperature; Table S6: Comparison of regression lines and ANOVA of thorax temperature; Table S7: Comparison of regression lines and ANOVA of abandoned comb temperature; Table S8: Comparison of regression lines and ANOVA of comb temperature (T_comb_) of *P. dominula* and *P. gallicus;* Table S9: Comparison of regression lines and ANOVA of thorax temperature (T_thorax_) of *P. dominula* and *P. gallicus*; Table S10: Comparison of regression lines and ANOVA of abandoned comb temperature (T_comb_) of *P. dominula* and *P. gallicus*. Figure S1: Typical habitat; Figure S2: Diurnal temperature variation; Figure S3: Comb temperature in relation to ambient nest temperature; Figure S4: Ambient nest temperature and head and abdomen temperature.
