# Supplementary material for: Relationship between Nest and Body Temperature and Microclimate in the Paper Wasp Polistes dominula
Source: Insects. 2023 Nov 16;14(11):886. doi: 10.3390/insects14110886 (PMC10672314; doi:10.3390/insects14110886)
Supplement: Supplementary file 1 [file insects-14-00886-s001.zip › insects-2646723-supplementary.pdf]

insects MDPI

Suppementary information

Relationship between Nest and Body Temperature and Microclimate in the Paper Wasp *Polistes dominula*

Helmut Kovac *, Julia Magdalena Nagy, Helmut Käfer and Anton Stabentheiner *

Institute of Biology, University of Graz, 8010 Graz, Austria
* Correspondence: helmut.kovac@uni-graz.at (H.K.); anton.stabentheiner@uni-graz.at (A.S.)

Table S1. Statistics for temperature measurements in inhabited and abandoned nests of *Polistes dominula* in Gschwendt (Austria) in the years 2017 and 2018.

Location	Date (d m y)	Nest	N_{cells}	N_{wasps}	Eggs	Larvae	Pupae	Nest	N_{cells}
		inhabited						abandoned	
Car tyre	28 June 2017	N5	205	14	✓	✓	✓		
Loft barn	1-2 July 2017	N1	70	5	✓	✓	✓	N1 ab.	36
Loft barn	5-6 July 2017	N2	61	8	✓	✓	✓	N2 ab.	44
Loft barn	7 July 2017	N3	120	15	✓	✓	✓	N3 ab.	35
Loft house	9 July 2017	N6	77	6	✓	✓	✓		
Car tyre	12 July 2017	N4	119	13	✓	✓	✓		
Loft barn	16-17 Aug 2017	N2	98	7	✓	✓	✓		
Loft barn	21-23 Aug 2017	N2	98	8	✓	✓	✓	N2 ab.	44
Loft barn	29-30 May 2018	N4	33	1	✓	✓	✓	N4 ab.	48
Loft barn	30-31 May 2018	N3	30	1	✓	✓	✓	N3 ab.	85
Loft barn	1-2 June 2018	N2	19	2	✓	✓	✓	N2 ab.	80
Loft house	3-5 June 2018	N1	70	4	✓	✓	✓	N1 ab.	101

Table S2. Statistics, fit functions and parameters for temperature measurements in inhabited and abandoned nests of *Polistes dominula* in Gschwendt (Austria) in the years 2017 and 2018.

Figure	Subject	Function	Parameter				R^2	P	N
			A	B	C	D			
Fig. 3a	T_{comb} inhabited	$y = A + B*x + C*x^2 + D*x^3$	9.05105	-0.51354	0.07808	-0.00116	0.98363	<0.0001	1715
	T_{comb} abandoned	$y = a + b*x$	-2.45437	1.14645			0.97405	<0.0001	1363
Fig. 3b	T_{thorax}	$y = A + B*x + C*x^2 + D*x^3$	5.49294	0.06197	0.04998	-7.40234E-4	0.98148	<0.0001	1647
Fig. 4a	spring	$y = A + B*x + C*x^2 + D*x^3$	6.43007	-0.02536	0.05187	-7.4764E-4	0.98682	<0.0001	685
	summer	$y = A + B*x + C*x^2 + D*x^3$	8.81949	-0.55165	0.08221	-0.00124	0.9827	<0.0001	1031
Fig. 4b	spring	$y = A + B*x + C*x^2 + D*x^3$	14.15161	-0.89171	0.08333	-0.00112	0.98132	<0.0001	616
	summer	$y = A + B*x + C*x^2 + D*x^3$	4.81327	0.10109	0.05005	-7.56456E-4	0.98457	<0.0001	1031
Fig. 5a	comb	$y = a + b*x$	8.49258	1.05446			0.90342	<0.0001	1502
Fig. 5b	thorax	$y = a + b*x$	8.36391	1.05546			0.90469	<0.0001	1435
Fig. 5c	air	$y = a + b*x$	8.30261	1.05339			0.89199	<0.0001	1502
Fig. 5d	comb	$y = a*b^x$	8.3212	1.0547			0.64273	<0.0001	1715
Fig. 5e	thorax	$y = a*b^x$	8.1602	1.05578			0.65873	<0.0001	1647
Fig. 5f	air	$y = a*b^x$	8.18374	1.05319			0.63651	<0.0001	1715
Fig. 6a	comb	$y = A + B*x + C*x^2 + D*x^3$	1.45637	1.05897	-0.00398	-3.46013E-5	0.9629	<0.0001	1715
Fig. 6b	thorax	$y = A + B*x + C*x^2 + D*x^3$	0.37233	1.26017	-0.01395	1.07677E-4	0.96026	<0.0001	1647
Fig. 6c	air	$y = A + B*x + C*x^2 + D*x^3$	-0.49319	1.45932	-0.02732	2.93958E-4	0.94532	<0.0001	1715
Fig. 7a	*P. dominula*								
	T_{comb} inhabited	$y = A + B*x + C*x^2 + D*x^3$	9.05105	-0.51354	0.07808	-0.00116	0.98363	<0.0001	1715
	T_{comb} abandoned	$y = a + b*x$	-2.45437	1.14645			0.97405	<0.0001	1363
	P. gallicus								
	T_{comb} inhabited	$y=A+(B-A)/(1+(x/c)^d)$	15.0000	39.84515	25.50334	5.3845	0.93453	<0.0001	748
	T_{comb} abandoned	$y = a + b*x$	0.89998	1.01364			0.93876	<0.0001	116
Fig. 7b	*P. dominula*	$y = A + B*x + C*x^2 + D*x^3$	5.49294	0.06197	0.04998	-7.40234E-4	0.97987	<0.0001	1647
	P. gallicus	$y = A + B*x + C*x^2 + D*x^3$	5.0000	0.03831	0.05254	0.93469	0.93835	<0.0001	748
Fig. S3	edge	$y = A + B*x + C*x^2 + D*x^3$	11.79205	-0.93881	0.09746	-0.00142	0.97763	<0.0001	1715
	center	$y = A + B*x + C*x^2 + D*x^3$	7.99135	-0.41099	0.07588	-0.00116	0.98007	<0.0001	1715
Fig. S4a	head	$y = A + B*x + C*x^2 + D*x^3$	7.54314	-0.22048	0.06197	-9.00655E-4	0.98208	<0.0001	1643
Fig. S4b	abdomen	$y = A + B*x + C*x^2 + D*x^3$	4.96596	0.14465	0.04595	-6.83109E-4	0.98598	<0.0001	1647

Table S3. Multiple linear regression model and ANOVA of comb temperature (T_{comb}) in relation to ambient air temperature at the nest ($T_a$nest), ambient air temperature of a standard meteorological weather station ($T_a$standard), substrate temperature ($T_{substrate}$), where the nest was attached to and to INCA weather data of GeoSphere Austria (T_aINCA).

Multiple Regression Analysis

Parameter	Estimation	Error	Statistics	P-value
Constant	1.28922	0.162866	7.91583	0
$T_a$nest	0.598943	0.016737	35.7856	0
$T_a$standard	0.320195	0.0171942	18.6223	0
$T_{substrate}$	0.205238	0.0113741	18.0444	0
T_aINCA	-0.112038	0.0124232	-9.01849	0

ANOVA

Source	Sum of Squares	Df	Mean Square	F-Ratio	P-value
$T_a$nest	76383.1	1	76383.1	83997.77	0
$T_a$standard	436.772	1	436.772	480.31	0
$T_{substrate}$	263.519	1	263.519	289.79	0
T_aINCA	73.9601	1	73.9601	81.33	0
Model	77157.4	4			

Table S4. Multiple linear regression model and ANOVA of thorax temperature (T_{thorax}) in relation to ambient air temperature at the nest ($T_a$nest), ambient air temperature of a standard meteorological weather station ($T_a$standard), substrate temperature ($T_{substrate}$), where the nest was attached to and to INCA weather data of GeoSphere Austria (T_aINCA).

Multiple Regression Analysis

Parameter	Estimation	Error	Statistics	P-value
Constant	0.702796	0.142562	4.92976	0
$T_a$nest	0.665755	0.0147225	45.2204	0
$T_a$standard	0.215165	0.0152285	14.1291	0
$T_{substrate}$	0.200059	0.0100039	19.9982	0
T_aINCA	-0.0474876	0.0110245	-4.30745	0

ANOVA

Source	Sum of Squares	Df	Mean Square	F-Ratio	P-value
$T_a$nest	75698	1	75698	110820.39	0
$T_a$standard	278.07	1	278.07	407.09	0
$T_{substrate}$	262.904	1	262.904	384.89	0
T_aINCA	12.6737	1	12.6737	18.55	0
Model	76251.7	4			

Table S5. Comparison of regression lines and ANOVA of comb temperature (T_{comb}) and isothermal line (T_{iso}: T_{comb} = $T_a$nest).

Multiple Regression Analysis

Parameter	Estimation	Error	Statistics	P-value
Constant	-0.416386	0.109417	-3.80548	0.0001
T_{comb}	1.06503	0.00441911	241.005	0
T_{comb}=T_{iso}	0.416386	0.265883	1.56605	0.1173
T_{comb}* T_{iso}	-0.0650281	0.00943133	-6.8949	0

Analysis of Variance

Source	Sum of Squares	Df	Mean Square	F-Ratio	P-value
Model	104833	3	34944.4	24163.99	0
Residuals	2618.95	1811	1.44613		
Total (Corr.)	107452	1814			

Further ANOVA

Source	Sum of Squares	Df	Mean Square	F-Ratio	P-value
T_{comb}	104627	1	104627	72349.6	0
Intersept	137.149	1	137.149	94.84	0
Slope	68.7487	1	68.7487	47.54	0
Model	104833	3			

Table S6. Comparison of regression lines and ANOVA of thorax temperature (T_{thorax}) and isothermal line (T_{iso}: T_{thorax} = T_{a}nest).

Multiple Regression Analysis

Parameter	Estimation	Error	Statistics	P-value
Constant	-0.65469	0.0958867	-6.82774	0
T_{thorax}	1.07913	0.00387947	278.165	0
T_{thorax}=T_{iso}	0.65469	0.22956	2.85194	0.0043
T_{thorax}* T_{iso}	-0.0791322	0.00815355	-9.70524	0

Analysis of Variance

Source	Sum of Squares	Df	Mean Square	F-Ratio	P-value
Model	103732	3	34577.3	32274.34	0
Residuals	1867.37	1743	1.07135		
Total (Corr.)	105599	1746			

Further ANOVA

Source	Sum of Squares	Df	Mean Square	F-Ratio	P-value
T_{thorax}	103467	1	103467	96576.3	0
Intersept	163.402	1	163.402	152.52	0
Slope	100.913	1	100.913	94.19	0
Model	103732	3			

Table S7. Comparison of regression lines and ANOVA of abandoned comb temperature (T_{comb}) and isothermal line (T_{iso}: T_{comb} = T_{a}nest).

Multiple Regression Analysis

Parameter	Estimation	Error	Statistics	P-value
Constant	-2.32416	0.106884	-21.7447	0
T_{comb}	1.14046	0.00453095	251.705	0
T_{comb}=T_{iso}	2.32416	0.243795	9.53325	0
T_{comb}* T_{iso}	-0.140462	0.00879144	-15.9771	0

Analysis of Variance

Source	Sum of Squares	Df	Mean Square	F-Ratio	P-value
Model	95983.7	3	31994.6	27059.47	0
Residuals	1902.45	1609	1.18238		
Total (Corr.)	97886.2	1612			

Further ANOVA

Source	Sum of Squares	Df	Mean Square	F-Ratio	P-value
T_{comb}	95563	1	95563	80822.59	0
Intersept	118.881	1	118.881	100.54	0
Slope	301.824	1	301.824	255.27	0
Model	95983.7	3			

Table S8. Comparison of regression lines and ANOVA of comb temperature (T_{comb}) of *P. dominula* and *P. gallicus*.

Multiple Regression Analysis

Parameter	Estimation	Error	Statistics	P-value
Constant	-0.416386	0.13463	-3.0928	0.002
T_{comb}	1.06503	0.00543741	195.871	0
P.dom.=P.gal.	9.00046	0.306846	29.3322	0
T_{comb} * Species	-0.31289	0.0102387	-30.5595	0

Analysis of Variance

Source	Sum of Squares	Df	Mean Square	F-Ratio	P-value
Model	126091	3	42030.4	19197.36	0
Residuals	5383.69	2459	2.18938		
Total (Corr.)	131475	2462			

Further ANOVA

Source	Sum of Squares	Df	Mean Square	F-Ratio	P-value
T_{comb}	124042	1	124042	56656.03	0
Intersept	4.71652	1	4.71652	2.15	0.1422
Slope	2044.63	1	2044.63	933.88	0
Model	126091	3			

Table S9. Comparison of regression lines and ANOVA of thorax temperature (T_{thorax}) of *P. dominula* and *P. gallicus*.

Multiple Regression Analysis

Parameter	Estimation	Error	Statistics	P-value
Constant	-0.65469	0.123319	-5.3089	0
T_{thorax}	1.07913	0.00498936	216.287	0
P.dom.=P.gal.	4.45491	0.277028	16.0811	0
T_{thorax} * Species	-0.153125	0.00926354	-16.5298	0

Analysis of Variance

Source	Sum of Squares	Df	Mean Square	F-Ratio	P-value
Model	137569	3	45856.3	25877.36	0
Residuals	4237	2391	1.77206		
Total (Corr.)	141806	2394			

Further ANOVA

Source	Sum of Squares	Df	Mean Square	F-Ratio	P-value
T_{thorax}	137085	1	137085	77358.83	0
Intersept	0.03112	1	0.03112	0.02	0.8946
Slope	484.19	1	484.19	273.24	0
Model	137569	3			

Table S10. Comparison of regression lines and ANOVA of abandoned comb temperature (T_{comb}) of *P. dominula* and *P. gallicus*.

Multiple Regression Analysis

Parameter	Estimation	Error	Statistics	P-value
Constant	-2.32416	0.109475	-21.23	0
T_{comb}	1.14046	0.0046408	245.747	0
P.dom.=P.gal.	3.24342	0.760752	4.26344	0
T_{comb} * Species	-0.127455	0.0272722	-4.67342	0

Analysis of Variance

Source	Sum of Squares	Df	Mean Square	F-Ratio	P-value
Model	79796.8	3	26598.9	21443.7	0
Residuals	2015.66	1625	1.24041		
Total (Corr.)	81812.5	1628			

Further ANOVA

Source	Sum of Squares	Df	Mean Square	F-Ratio	P-value
T_{comb}	79761.9	1	79761.9	64302.97	0
Intersept	7.78222	1	7.78222	6.27	0.0123
Slope	27.0915	1	27.0915	21.84	0
Model	79796.8	3			

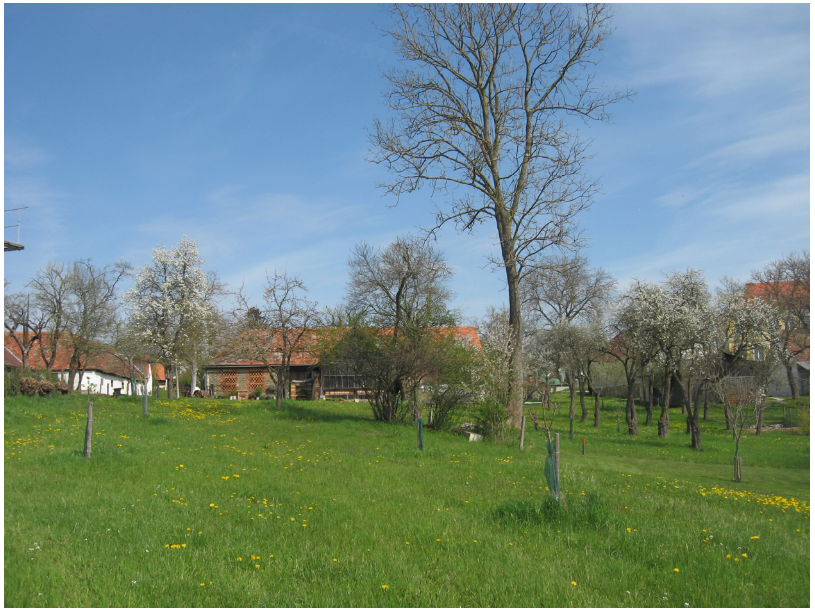

Figure S1. Typical habitat of the paper wasp *Polistes dominula* and research location in a in a rural area in Gschwendt (Styria, Austria) in a temperate climate region of Cetral Europe.

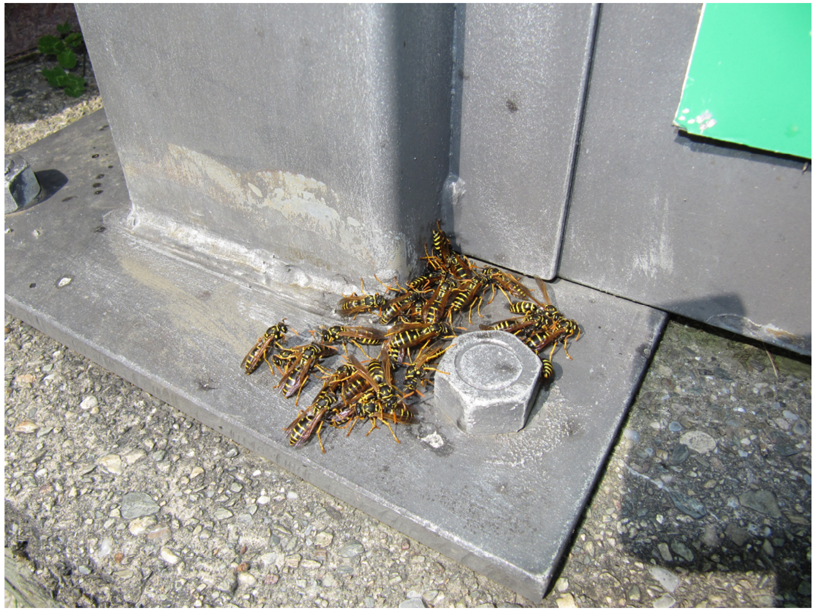

Figure S2. Man-made breeding location of the paper wasp *Polistes dominula* at 1160 m ASL in an Alpine climate region in Obergail (Lesachtal, Carinthia, Austria), 16 Sept. 2019.

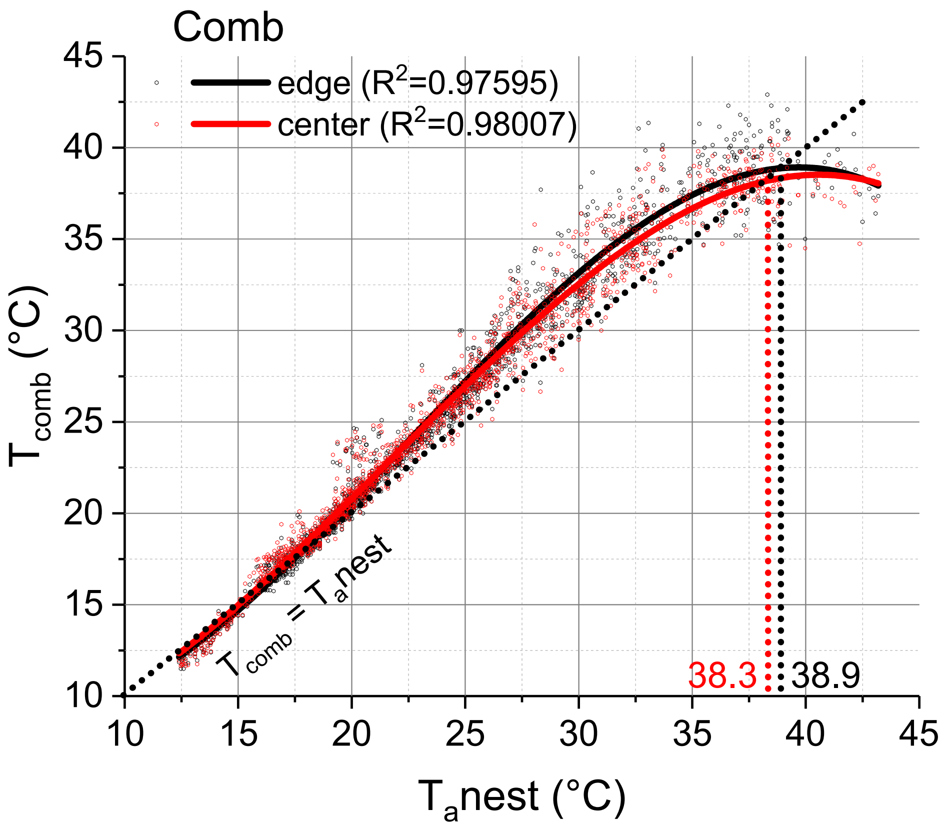

Figure S3. Comb temperature (T_{comb}) in relation to ambient nest temperature ($T_a$nest). Temperature curves from cells the edge and in the center of the comb, points of intersection with isothermal line (dotted line indicates a slope of 1, T_{comb} = $T_a$nest) are indicated by vertical dotted line.

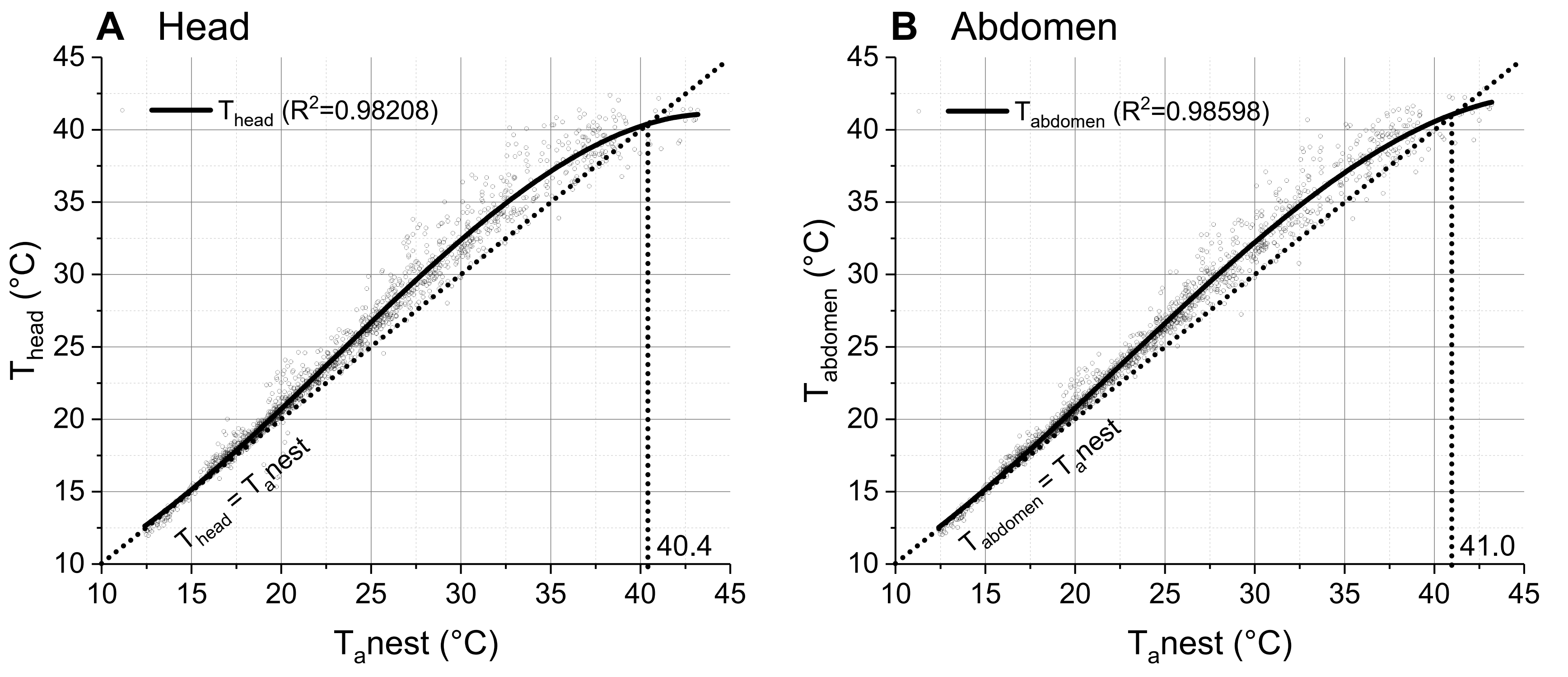

Figure S4. Body temperature in relation to ambient nest temperature ($T_a$nest): (A) Head (T_{head}) and (B) abdomen ($T_{abdomen}$) temperature of wasps from inhabited nests, point of intersection with isothermal line ($T_{head/abdomen}$ = $T_a$nest) is indicated by vertical dotted line.
